# TB and primary health care: a synergistic pathway to universal health coverage

**DOI:** 10.5588/ijtldopen.25.0611

**Published:** 2026-01-09

**Authors:** D. Pedrazzoli, F. Khalid, F. Mavhunga, T. Kasaeva, S.B. Syed, T. Islam

**Affiliations:** World Health Organisation, 20 Avenue Appia,1211 Geneva, Switzerland.

**Keywords:** tuberculosis, End TB Strategy, primary health care, UHC

## Abstract

TB remains a major global health challenge, disproportionately affecting marginalized populations and exposing inequities. The WHO End TB Strategy aligns with the primary health care (PHC) approach to advance universal health coverage (UHC) and sustainable outcomes. Drawing on WHO’s recent policy brief ‘Tuberculosis and primary health care: synergies and opportunities towards universal health coverage’, this article highlights the importance of reorienting health systems towards PHC to strengthen TB responses through integrated services, multisectoral collaboration and empowered communities. It explores synergies between PHC and the End TB Strategy, presenting strategies, barriers, and recommendations to promote equity, efficiency, and resilience, to accelerate progress toward ending TB.

TB continues to cast a long shadow over global health, with an estimated 1.3 million deaths and nearly 11 million cases in 2023, about 3 million of which were undiagnosed or unreported.^1^ TB primarily affects individuals of all ages. Around 30% of TB cases are linked to key risk factors such as undernutrition, diabetes, smoking, HIV, and alcohol use.^1^ Even after treatment, people often face long-term impairments, including reduced lung function and mental health challenges,^2^ underscoring the need for integrated, life-course TB care. TB persists largely because of socioeconomic inequities.^3^ Over 80% of cases occur in low- and middle-income countries, where poverty, food insecurity, overcrowded housing, and poor access to care increase vulnerability.^1^ Stigma continues to hinder diagnosis and treatment, further compounding these challenges and underscoring the need for people-centred, community-based care.^4^ Addressing TB therefore requires health system action alongside broader social interventions.^5^

The WHO End TB Strategy calls for people-centred prevention and care, health system sustainability, and action on social determinants.^6^ Rooted in the goal of universal health coverage (UHC), it aligns with WHO’s promotion of primary health care (PHC) as the main pathway to UHC and the Sustainable Development Goals (SDGs).^7^ PHC integrates service delivery, multisectoral policy, and empowered communities, making it a transformative framework for strengthening TB responses.^8,9^ The 2023 UN High-Level Meetings on TB and UHC reaffirmed PHC as the foundation for ending TB.^10,11^ WHO’s recent policy brief underscores the need to reorient health systems toward PHC to accelerate progress.^12^ Doing so will both strengthen TB responses and enhance resilience against broader health challenges, as demonstrated during COVID-19.^13,14^ As global health systems face the dual challenges of post-pandemic recovery and tightening fiscal constraints, the imperative to deliver more equitable, integrated, and sustainable health services has never been more urgent.^15^

## SYNERGIES BETWEEN PHC AND THE END TB STRATEGY

PHC represents a whole-of-society approach that integrates health services, engages multiple sectors, and empowers communities.^8^ Applied to TB, this means embedding prevention, diagnosis, treatment, and long-term care in PHC systems, ensuring both medical and social needs are addressed. The End TB Strategy reflects PHC principles. Pillar 1: Integrated, people-centred care links directly with PHC’s service delivery; Pillar 2: Bold policies and supportive systems align with PHC’s focus on governance, financing, and multisectoral collaboration; Pillar 3: Intensified research and innovation support PHC-oriented tools.^6,16^ Together, these synergies provide sustainable platforms to advance UHC while tackling TB.

### 1. Integrated service delivery

Reorienting TB services through PHC means decentralizing diagnosis, treatment, and prevention to primary care facilities. Integration allows comorbidities such as HIV, diabetes, and undernutrition to be managed within the same care pathways, improving efficiency and outcomes. PHC also enables comprehensive, life-course service packages, improving continuity and reducing duplication. Such integration supports accessibility and strengthens trust in health systems.

### 2. Multisectoral policy and action

TB is shaped by poverty, malnutrition, and housing conditions, so reorientation must involve partnerships across health, education, housing, and labour sectors. The WHO Multisectoral Accountability Framework for TB (MAF-TB) offers a model for coordinating policies, aligning actions, and ensuring accountability.^17^ PHC helps operationalize these approaches, fostering governance mechanisms that integrate TB and broader health system priorities.

### 3. Empowered communities

Community empowerment is central to PHC and essential for ending TB. Civil society and community health workers play critical roles in co-designing services, reducing stigma, and supporting treatment adherence.^18^ Embedding community participation in governance ensures services reflect people’s needs, while fostering advocacy and accountability. Empowered communities strengthen service demand, monitor delivery, and sustain political and financial commitment.^18^

## OPPORTUNITIES AND LEVERS FOR REORIENTATION

Reorienting health systems toward PHC in the fight against TB requires coordinated action across several levers:

- Governance and leadership: political commitment is crucial to align TB programmes with health system strengthening. Strong governance fosters accountability and inclusive decision-making.^9^
- Financing: sustainable financing is vital to transition from disease-specific programmes to integrated PHC services. Innovative approaches such as pooled or blended financing can mobilize resources for both TB and broader health goals.^15,19,20^
- Community engagement: active participation by affected communities reduces stigma, enhances adherence, and improves service delivery.^18^
- Capacity and infrastructure: investments are needed in workforce training, infrastructure, and institutional capacity to deliver integrated, decentralized TB care. Multidisciplinary teams trained in people-centred care can ensure sustainability.^9^

## THE WAY FORWARD

Given the variability in health system maturity, TB programme development, and socioeconomic contexts across countries, a shift to PHC-oriented health systems requires a nuanced, context-sensitive approach.^12^ Targeted strategies must be developed through comprehensive contextual analyses that identify entry points for aligning national health systems with PHC principles, particularly in relation to TB prevention, diagnosis, treatment and long-term care. Countries with strong PHC foundations should focus on improving service quality, governance and integration of TB within broader care models. In contrast, countries with nascent PHC systems may need to prioritise infrastructure, essential service packages, workforce training and community participation. Rigorous assessments of national health and TB landscapes are required to guide strategies tailored to local contexts. Operationalizing this shift involves linked actions: defining service packages, strengthening accountability frameworks, and embedding TB services within PHC pathways (see Table). Robust monitoring and evaluation are essential. Indicators might include the share of TB services delivered at the primary care level, access to diagnostics and medicines, service coverage, and community satisfaction. These measures allow continuous learning, refinement, and accountability.

**Table.** Key enablers for advancing PHC-oriented health systems in the context of TB.

**Key enabler**	**Description**
**Coordinated planning**	Align national TB strategic plans with global frameworks (e.g., End TB Strategy), adapting key principles and targets to the local health system context. Promote coordinated planning to strengthen integrated service delivery and reduce fragmentation in line with the PHC approach.
**Policy dialogue for system designing**	Strengthen the PHC approach to TB through ongoing policy dialogue and advocacy. Adapt service design, prioritization, funding, and delivery to meet people’s comprehensive, life-course needs. A systems focus supports broader health outcomes and disease-specific targets.
**Streamlined health financing and sustainable funding**	Mobilise and allocate sustainable financing which is critical to supporting the transformation of health systems into PHC-oriented models that can deliver comprehensive TB services.
**Systems thinking and change management^21^**	Apply systems thinking to understand and address the complexity of health systems, enhancing PHC and TB responses. Promote effective change management through strategic communication, people-centred dialogue, and advocacy to drive policy shifts and improve health outcomes.
**Defined TB service packages at different levels of the health system**	Include TB in the national package of essential health services. Define and standardize TB care across different levels of the health system to guide PHC design and ensure consistent, appropriate service delivery.
**Health workforce, infrastructure and equipment**	Strengthen PHC-oriented TB care by ensuring a well-distributed, trained, motivated, and supported health workforce. Recognize and support community health workers. Ensure regular capacity-building and provide adequate infrastructure, equipment, and supplies to maintain service quality at all levels.
**Implementation research and sharing country experiences**	Conduct implementation research to inform policy, guide PHC-oriented reforms, and support scale-up of TB services. Share country experiences and lessons learned to foster experiential learning and strengthen PHC efforts globally.

## CONCLUSION

Reorienting health systems toward PHC provides a transformative opportunity to end TB, achieve UHC, and advance the SDGs. This requires sustained commitment from governments, civil society, and international stakeholders to integrate PHC principles into health system reforms. Multisectoral collaboration, robust financing, and empowered communities will be critical. PHC-oriented reconfiguration strengthens both TB responses and wider health systems, ensuring equity, resilience, and sustainability. By embedding PHC as the backbone of health systems, countries can address the root causes of vulnerability and inequity while delivering people-centred services. Achieving this vision demands decisive action but promises a healthier and more equitable future where no one is left behind.
